# Long term usage of dexamethasone accelerating the initiation of osteoarthritis via enhancing the extracellular matrix calcification and apoptosis of chondrocytes

**DOI:** 10.7150/ijbs.64152

**Published:** 2021-10-03

**Authors:** Liang Chen, Zhenhong Ni, Junlan Huang, Ruobin Zhang, Jinfan Zhang, Bin Zhang, Liang Kuang, Xianding Sun, Dali Zhang, Nan Su, Huabing Qi, Jing Yang, Min Jin, Fengtao Luo, Hangang Chen, Siru Zhou, Xiaolan Du, Junjie Ouyang, Zuqiang Wang, Yangli Xie, Qiaoyan Tan, Lin Chen

**Affiliations:** 1 Center of Bone Metabolism and Repair, Department of Wound Repair and Rehabilitation Medicine, State Key Laboratory of Trauma, Burns and Combined Injury, Trauma Center, Research Institute of Surgery, Daping Hospital, Army Medical University, Chongqing, China.; 2 Department of orthopedic, Daping Hospital, Army Medical University, Chongqing, China.

**Keywords:** dexamethasone, articular cartilage, calcification, apoptosis, AKT

## Abstract

Systemic application of glucocorticoids is an essential anti-inflammatory and immune-modulating therapy for severe inflammatory or autoimmunity conditions. However, its long-term effects on articular cartilage of patients' health need to be further investigated. In this study, we studied the effects of dexamethasone (Dex) on the homeostasis of articular cartilage and the progress of destabilization of medial meniscus (DMM)-induced osteoarthritis (OA) in adult mice. Long-term administration of Dex aggravates the proteoglycan loss of articular cartilage and drastically accelerates cartilage degeneration under surgically induced OA conditions. In addition, Dex increases calcium content in calcified cartilage layer of mice and the samples from OA patients with a history of long-term Dex treatment. Moreover, long term usage of Dex results in decrease subchondral bone mass and bone density. Further studies showed that Dex leads to calcification of extracellular matrix of chondrocytes partially through activation of AKT, as well as promotes apoptosis of chondrocytes in calcified cartilage layer. Besides, Dex weakens the stress-response autophagy with the passage of time. Taken together, our data indicate that long-term application of Dex may predispose patients to OA and or even accelerate the OA disease progression development of OA patients.

## Introduction

Glucocorticoids 1 have been systemically used clinically as common anti-inflammatory and immunosuppressive drugs for severe inflammatory or autoimmunity conditions. About 20% of all glucocorticoid medications are long-term usage 2, which have been applied in 1% to 3% of the adults in the world's adults 3. Prolonged treatment with glucocorticoid can cause severe complications such as insulin resistance, atrophies of muscle and skin, depression, and severe effects on the skeletal system, *etc*. Numerous reports have indicated that systemically administered glucocorticoids have adverse effects on development and homeostasis of skeletal system, including impairment of skeleton development and glucocorticoid-induced osteonecrosis and osteoporosis 4-7.

Osteoarthritis (OA) is one of the most prevalent chronic joint diseases with progressive cartilage destruction and insufficient extracellular matrix synthesis 8. Dexamethasone (Dex), as the most potent member of the glucocorticoid's family 9. Previous research has demonstrated that Dex can rescue cartilage matrix loss and chondrocyte viability in animal studies and cartilage explant models of tissue injury and post-traumatic OA 10. However, some studies suggested that Dex has adverse effect on the developing cartilage. Glade *et al.* 11 found that daily intramuscular Dex injection led to degenerative joint disease in growing foals evidenced by cartilage destruction including fissures and avulsions in the periarticular cartilage. Likewise, Tomaszewska *et al.* 12 found that prenatal Dex treatment resulted in an overall reduction of articular cartilage of piglets. Moreover, some evidence also suggested the side effect of glucocorticoids on adult cartilage. Degenerative changes of the articular cartilage of adult rats after Dex administration were demonstrated by electron microscopy 13, 14.

Dex has been reported to have differential effect on cartilage depending on the dose and duration of its exposure as well as the model systems used. It is crucially important to understand under what conditions Dex may be beneficial or harmful to articular cartilage, which will help to optimize the safe use of this glucocorticoid in the clinic as an anti-inflammatory and immunosuppressive drug. Little is known, however, regarding the *in vivo* effect of the long term usage of Dex on articular cartilage*.* So, the effects of systemic application of Dex on the maintenance of articular cartilage or even the progress of OA need to be further investigated.

Here we used mouse model to dissect the adverse effect of Dex on healthy and osteoarthritic articular cartilage. Our study revealed that long term usage of Dex aggravates the proteoglycan loss of articular cartilage and accelerates the progression of OA *in vivo*. Further mechanism study elucidated that Dex plays a critical role in accelerating the initiation of OA through enhancing the extracellular matrix calcification of chondrocytes via activation of AKT signaling as well as increasing chondrocyte apoptosis. Our results clearly demonstrated that long term application of Dex may predispose patients to OA or even accelerate the OA pathogenesis of patients, which need to be considered by the patients and clinicians during their management of diseases related.

## Materials and methods

### Surgical model of OA in mice and Dex administration

10-week-old male C57BL/6J mice (25-30 g) were purchased from the Beijing HFK Bioscience Co. Ltd. All mice maintained in the animal facility of the Daping Hospital (Chongqing, China). Animal experiments were performed according to protocols approved by the Laboratory Animal Welfare and Ethics Committee of the Army Medical University (Chongqing, China). The mice were randomly divided into six groups: Control groups (vehicle group and Dex group), Sham groups (Sham + vehicle group, Sham + Dex group), and destabilization of medial meniscus (DMM) groups (DMM + vehicle group, DMM + Dex group) (n = 8). DMM surgery was performed on the right knee joints to establish experimental OA model according to the described methods according to previous protocol 15, sham surgery was performed with medial capsulotomy only on the left knee joints of 12-week-old male mice. After 2 days, Dex was used according to the previous studies 16, 17. Briefly, mice in treatment group received Dex (5 mg/kg, 3 times per week) postoperatively via intraperitoneal injection for 4, 8 and 12 weeks. The mice in vehicle group were injected intraperitoneally with saline only.

### Histologic assessment

The knee joints were fixed with 4% paraformaldehyde for 24 h, decalcified with 0.5 M EDTA at pH7.4 for 2 weeks and embedded in paraffin. Five-micrometer-thick sagittal serial sections were made across the knee joints. Histologic grading of cartilage degeneration was performed using the OARSI recommended subjective scoring system (on a scale of 0-6), cartilage proteoglycan depletion was scored (on a scale of 0-5) as a complement measure of cartilage degeneration 18.

### Methyl methacrylate embedding and sectioning

The undecalcified specimens were dehydrated in ascending concentrations of ethanol (from 70% to 100%) and then embedded in methyl methacrylate according to the instructions of manufacturer. The embedded specimens were trimmed with a hard tissue cutting device (Leica RM2265; Germany) to expose the target area. Finally, the cut specimens were sanded down with sequential #2000, #4000, #8000 and #10000 grit lapping plastic sandpaper (3M; Japan). Each specimen was placed in an ultrasonic bath after the polishing steps.

### Scanning electron microscope imaging and energy dispersive spectrometer detection

According to previous protocol 19, after vacuum drying, the samples were sputter-coated with gold and then observed by scanning electron microscope (SEM, SU8010, Hitachi, Japan) under 5 keV accelerating voltage, 10 μA probe current, a 10-mm working distance, and an image resolution of 1560 × 1920. The calcium element composition was analyzed using an energy dispersive spectrometer (EDS, Oxford X-max50, Oxford, UK).

### Sample collection and processing

This study was conducted in approval of ethics committee of the Daping Hospital (Chongqing, China). In OA group, human articular cartilage samples were collected from 3 primary knee OA (grade IV in The Kellgren Lawrence grading system) patients with multiple intra-articular glucocorticoid injections and Varus deformity receiving knee arthroplasty. In control group, human articular cartilage samples were collected from 3 patients who had amputations due to trauma without inflammatory arthritis or prior knee surgery history. All samples of full thickness articular cartilage were cut from patient's tibial plateau and fixed in 4% PFA. Half of them were decalcified in 15% EDTA for wax embedding, and remaining samples underwent hard tissue embedding.

### Microcomputed tomography (micro-CT)

Undecalcified specimens were scanned by the vivaCT 40 micro-CT system (Scanco Medical, Brüttisellen, Switzerland) with the settings of 70 kV and 113 mA. Constant thresholds 212 were applied to grayscale images to distinguish bone from soft tissue.

### Isolation and culture of primary chondrocyte

Primary chondrocytes from mouse growth plate were isolated and cultured according to our previous report 20. Briefly, the cartilage was isolated from knee joints of 3~5-day-old C57BL/6J mice followed by 15 min digestion with 0.25% trypsin at 37 °C in a CO2 incubator, then soft tissues (including muscles, ligaments and bone tissues) were carefully excised from their attachment sites by a surgical knife under stereomicroscope. Further incubation with 0.1% type II collagenase (Gibco, 17101-015) overnight at 37 °C in a CO2 incubator. Chondrocytes were cultured in DMEM/F12 (1:1), supplemented with 10% FBS. Chondrocytes were treated with Baf-A1 (10 nM, Selleck, S1413), an inhibitor of autophagy, with/without Dex for 24 h. Cell apoptosis was detected by TUNEL assay according to the manufacturer's instructions.

### Calcification analysis

Cells were cultured for 7 days in BGJb medium (Fitton-Jackson Modification, Gibco, 12591038) (plus 10% FBS, 50 mg/mL ascorbic acid, 20 mM β-glycerol phosphate), stimulated with Dex (0, 1, 10 and 100 nM) based on previous literature report 21, and with/without AKT inhibitor: LY294002 (10 μM, MedChemExpress, 154447-36-6). Medium was changed every 4 days. Alizarin red staining and Alizarin red absorbance at 562 nm were used to evaluate the degree of calcification.

### Western blotting

Protein was extracted using ice-cold RIPA lysis buffer (Beyotime, P0013B) containing protease inhibitors (Roche, 5892970001). Equal amount of protein samples was resolved by 10% SDS-PAGE gel and transferred onto polyvinylidene difluoride membrane. After blocking with 8% nonfat milk, the membrane was probed with antibody specific to AKT (1:1000 dilution; Cell Signaling Technology, #9272), P-AKT^308^ (1:1000 dilution; Cell Signaling Technology, #13038), P-AKT^473^ (1:1000 dilution; Cell Signaling Technology, #9271) and β-Actin (1:10000 dilution; Sigma-Aldrich, A1978) followed by chemiluminescent (Pierce, 34080) detection.

### Immunohistochemical staining (IHC)

IHC were performed with the SP-9000 Histostain-Plus kits (ZSGB-BIO) as previous protocols. The following antibodies were used: LC3B (1:200 dilution; Cell Signaling Technology, #2775), P-AKT^308^ (1:500 dilution; Cell Signaling Technology, #13038) followed by diaminobenzidine (DAB) kit detection. Positive cells from three random high-power fields were calculated for statistical analysis.

### TUNEL assay

Apoptotic cells in articular cartilage were detected by Click-iT™ TUNEL Alexa Fluor™ 647 (Thermo Fisher Scientific, C10247) according to the manufacturer's instructions. Images were observed and photographed under a laser scanning confocal microscope (ZEISS, LSM 880). The number of apoptotic chondrocytes in relation to the total number of cells was quantified in tissue sections. More than three fields of microscopic view in each section, and multiple sections (more than three) from 4 different experimental animals in each experimental group were used.

### Statistical analysis

Statistical analysis was performed using GraphPad Prism (GraphPad, La Jolla, CA). All numerical values were presented as mean values ± the standard error of mean (SEM) or mean values the standard deviation (SD). Student's t-test or ANOVA analysis followed by Tukey's post hoc tests were used to determine significance. Difference was considered significant when p value less than 0.05.

## Results

### Long term usage of Dex aggravates the proteoglycan content loss of articular cartilage in mice

**Figure 1:**
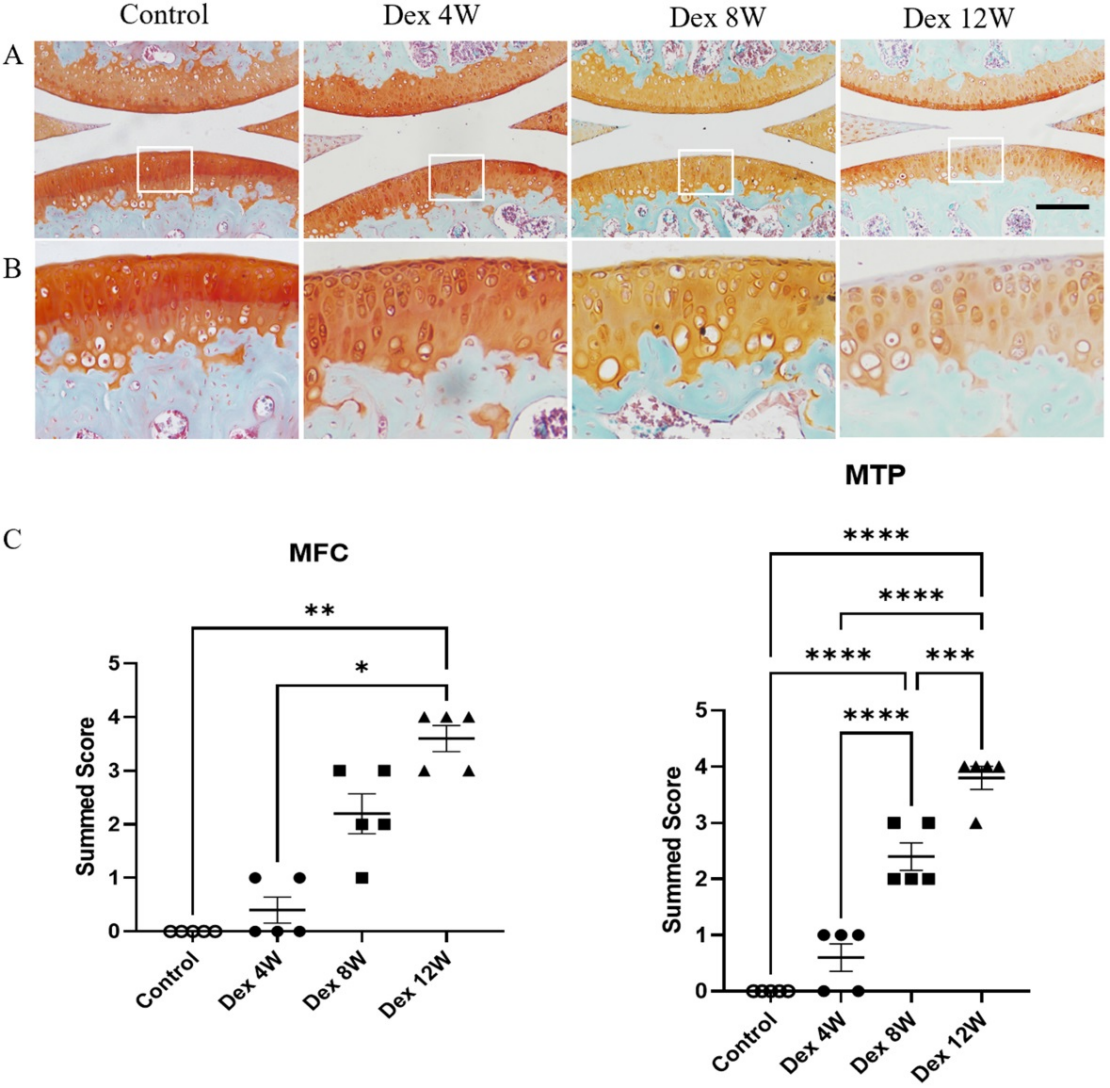
** Histologic features of mouse articular cartilage at 4, 8 and 12 weeks following long term usage of Dex. (A)** Representative images of Safranin O-fast green-stained sections of knee joints from intraperitoneal Dex injection mice at 4, 8 and 12 weeks. **(B)** Expansion of the region occupied by articular cartilage. **(C)** OARSI scoring system showed more severe proteoglycan depletion in the medial femur and tibia of mice following long term usage of Dex with advancing time (n = 5). MFC: medial femoral condyle; MTP: medial tibial plateau. Scale bar: 100 µm. Comparisons of multiple groups were evaluated using analysis of variance (ANOVA) followed by Tukey's test. Data were expressed as the mean ± SEM. *, P < 0.05; **, P < 0.01; ***, P < 0.001; ****, P < 0.0001.

Firstly, we studied the effect of Dex on the articular cartilage of mice. The mice were sacrificed at 4, 8 and 12 weeks after repeated Dex treatment. Then the knee joint samples were obtained and stained with Safranin O-fast green to assess the extent of articular cartilage degeneration by cartilage proteoglycan depletion scoring of OARSI on a scale of 0-5. Histologic assessment showed that the loss of extracellular matrix of articular cartilage was progressively aggravated in Dex treated mice from 8 to 12 weeks (Figure 1A). Accordingly, the OARSI scores of proteoglycan loss were significantly increased after Dex treatment mice from 8 to 12 weeks compared to those in control mice (Figure 1B). These data indicate that long term usage of Dex aggravates the loss of proteoglycan content of articular cartilage in mice.

### Long term usage of Dex exacerbates the pathological severity in experimental mouse OA model

**Figure 2:**
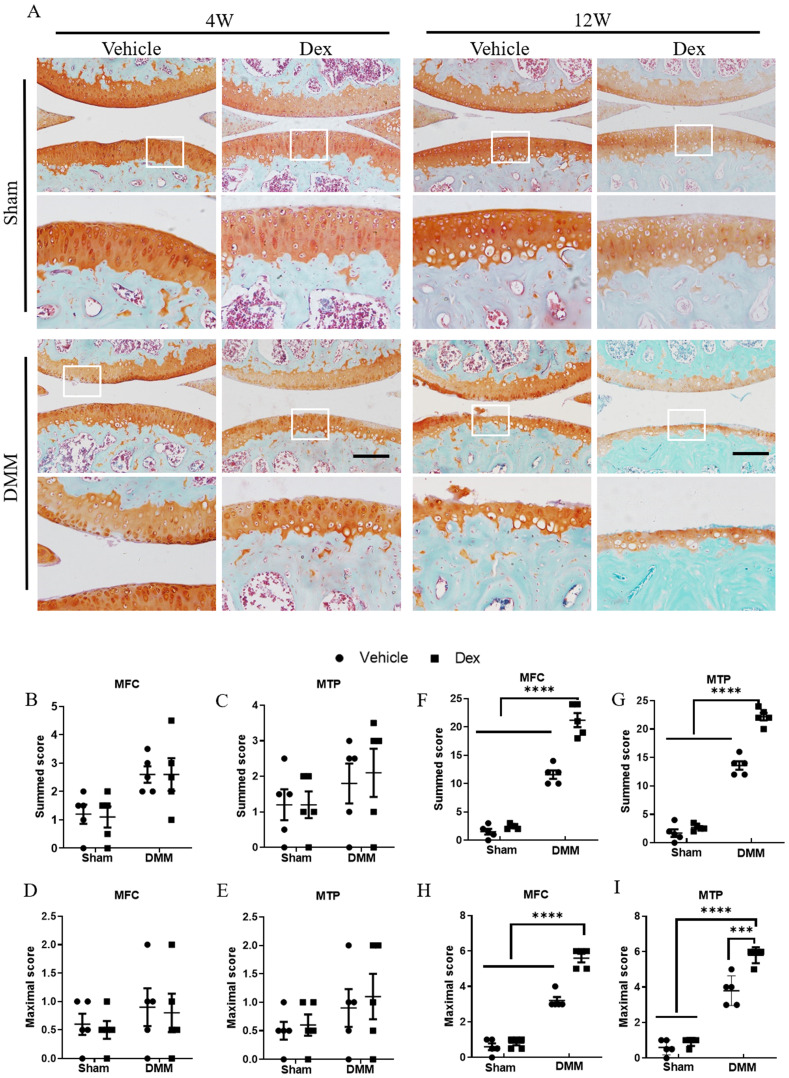
** Histologic analysis of structural damage in the articular cartilage following long term usage of Dex in experimental OA model. (A)** The articular cartilage was stained with Safranin O-fast green at 4 and 12 weeks after DMM surgery following long term usage of Dex to assess the extent of articular cartilage degeneration, expansion of the region occupied by articular cartilage. **(B-E)** OARSI scoring system showed intraperitoneal Dex injection exacerbates the pathological severity of articular cartilage at 4 weeks after DMM surgery (n = 5). **(F-I)** OARSI scoring system showed intraperitoneal Dex injection exacerbates the pathological severity of articular cartilage at 12 weeks after DMM surgery (n = 5). MFC: medial femoral condyle; MTP: medial tibial plateau. Scale bar: 100 µm. Comparisons of multiple groups were evaluated using analysis of variance (ANOVA) followed by Tukey's test. Data were expressed as the mean ± SEM. *, P < 0.05; **, P < 0.01; ***, P < 0.001; ****, P < 0.0001.

Next, to evaluate the effect of long-term usage of Dex on the cartilage damage in experimental OA model, we performed DMM surgery in the right knee joints of 12-week-old mice. After long term usage of Dex, histologic examination at 4 weeks after DMM surgery showed an early OA-like manifestations including loss of proteoglycan content of articular cartilage (Figure 2A-E). Consistently, the OA-like phenotype became more profound in mice with deficiency of large areas of surface articular cartilage and higher OARSI scores (Figure 2A, F-I). These findings suggest that long term usage of Dex exacerbates the pathological severity in OA mice.

### Dex increases calcium content in calcified cartilage layer (CCL)

**Figure 3:**
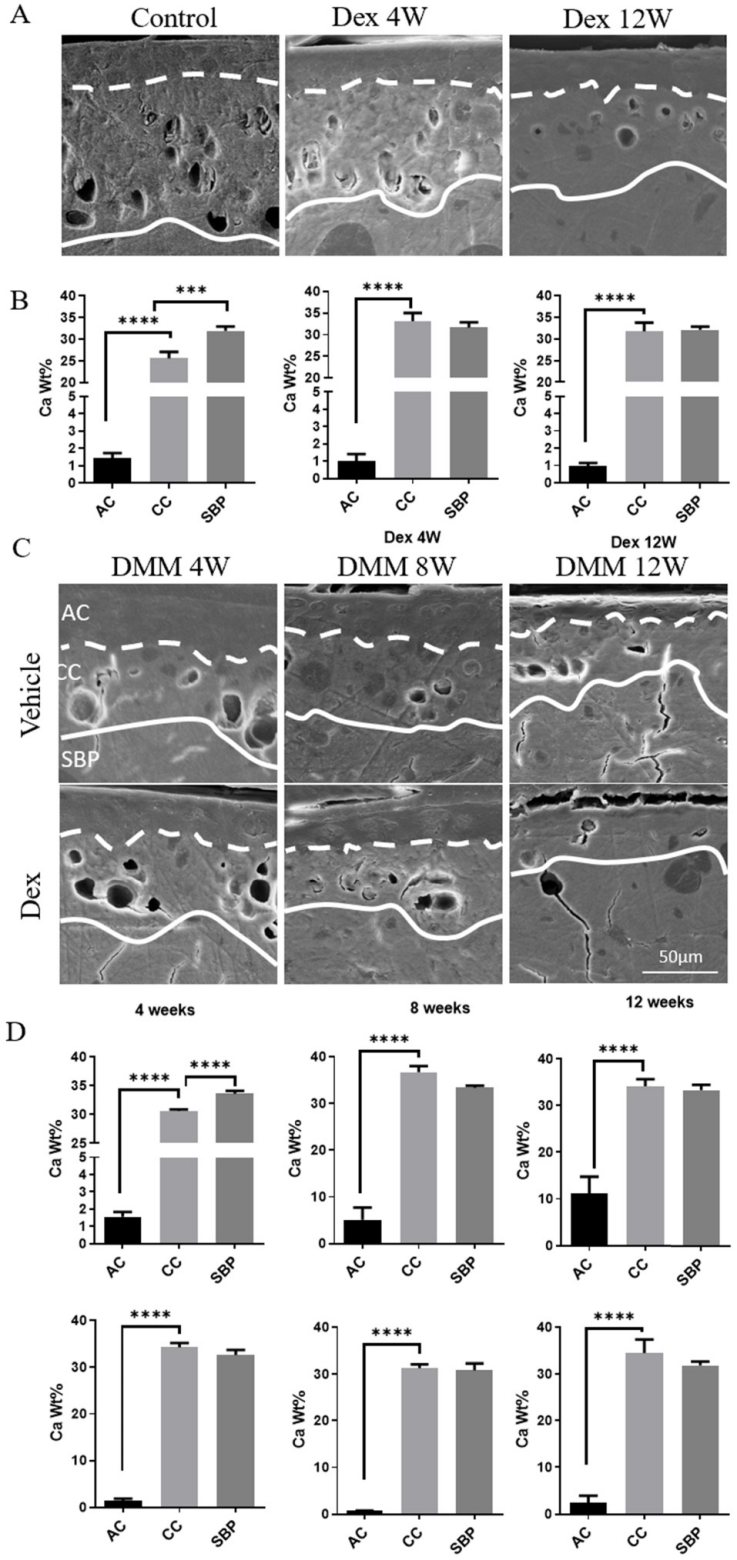
** Calcium content analysis of articular cartilage in normal and experimental OA model following long term usage of Dex. (A)** Representative images of scanning electron microscopy of articular cartilage with or without Dex. **(B)** Calcium content in each layer of articular cartilage (non-calcified cartilage layer: AC, calcified cartilage layer: CC, subchondral bone plate: SBP) was determined by energy dispersive spectrometer analysis at 4 and 12 weeks compared with control group with intraperitoneal injection of Dex (n = 3). **(C)** Representative images of scanning electron microscopy of articular cartilage at 4, 8 and 12 weeks after DMM surgery with or without Dex. **(D)** Calcium content in each layer of articular cartilage (non-calcified cartilage layer: AC, calcified cartilage layer: CC, subchondral bone plate: SBP) was determined by energy dispersive spectrometer analysis at 4, 8 and 12 weeks after DMM surgery with or without Dex (n = 3). Scale bar: 50 µm. Comparisons of multiple groups were evaluated using analysis of variance (ANOVA) followed by Tukey's test. Data were expressed as the mean ± SD. *, P < 0.05; **, P < 0.01; ***, P < 0.001; ****, P < 0.0001.

**Figure 4:**
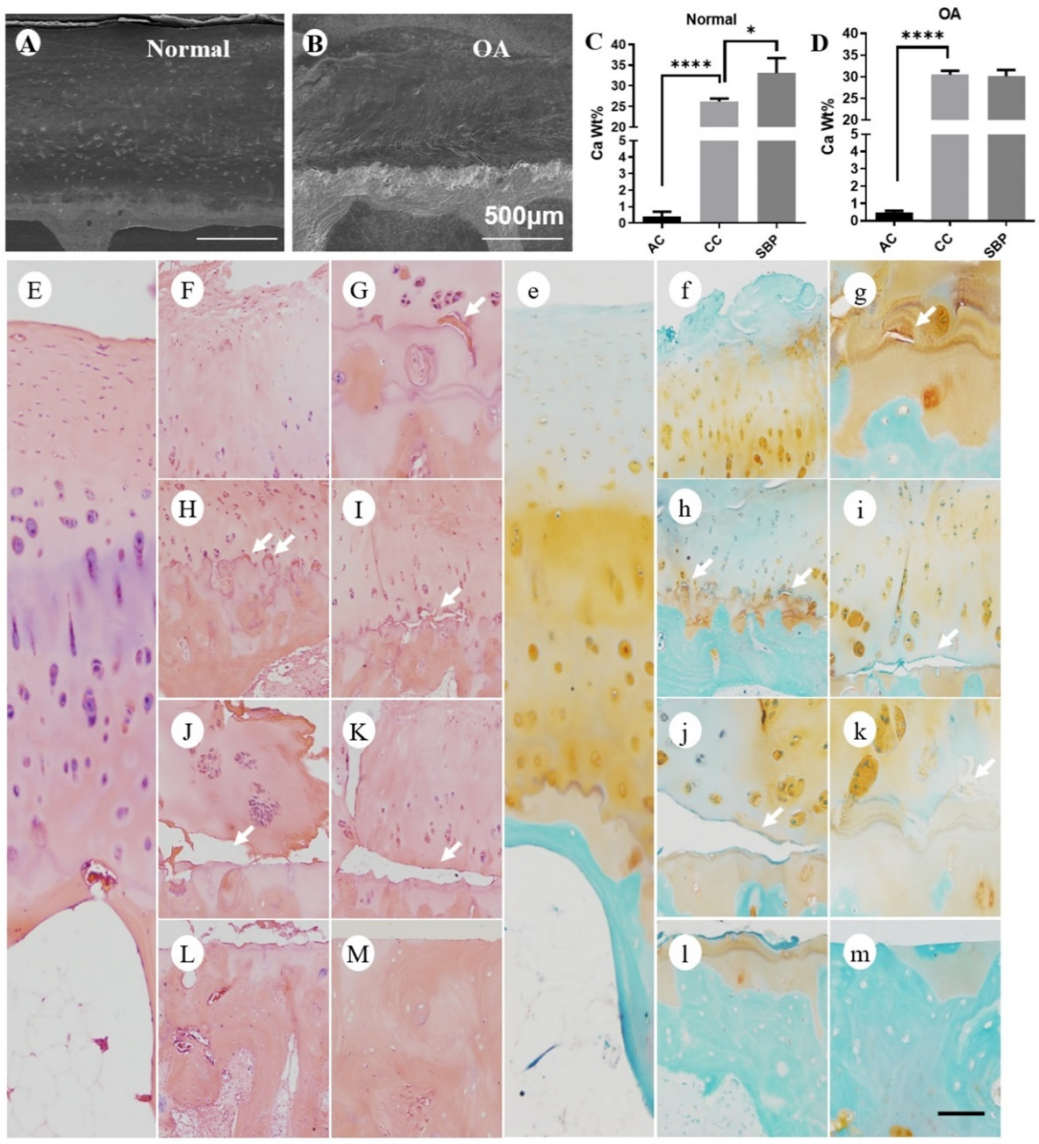
** Calcium content and histologic analysis of articular cartilage in normal and OA patients with a history of long-term usage of Dex. (A-B)** Representative images of scanning electron microscopy of articular cartilage in normal (A) and OA (B) patients. **(C-D)** Calcium content in each layer of articular cartilage (non-calcified cartilage layer: AC, calcified cartilage layer: CC, subchondral bone plate: SBP) was determined by energy dispersive spectrometer in normal (C) and OA (D) patients (n = 3). Scale bar: 500 µm. Comparisons of multiple groups were evaluated using analysis of variance (ANOVA) followed by Tukey's test. Data were expressed as the mean ± SD. *, P < 0.05; **, P < 0.01; ***, P < 0.001; ****, P < 0. 0001. The articular cartilage was stained with H & E (**E-M**) and Safranin O-fast green (e-m) in normal (E and e) and OA (F-M and f-m) patients (n = 3). Lots of horizontal clefts (arrowheads) can be found in sections of OA patients' samples. Scale bar: 100 µm.

The calcification of cartilage is highly involved in the pathological changes of OA 22. To further investigate the mechanisms of Dex-worsened cartilage damage, we detected the calcium content in each layer of articular cartilage of mouse knee joint by SEM and EDS. There were significant calcium concentration gradients in each layer of cartilage in the knee joint of normal adult mice (Figure 3A-B). The calcium content in the non-calcified cartilage layer was extremely low, while it increased successively in the calcified cartilage and subchondral bone plate (Figure 3B). The abnormally increased calcium content in the CCL was detected from 4 weeks to 12 weeks after Dex treatment (Figure 3B), which disturbed the original calcium distribution pattern. Next, we assessed the changes of cartilage calcification in DMM model (Figure 3C-D). We found that the calcium content in CCL was gradually increased during the OA process (from 8 to 12 weeks). Dex group, however, showed a suddenly abnormal increase of calcium content in the CCL at the early period (4 weeks). Moreover, the samples from OA patients with a history of Dex treatment presented a high calcium content in calcified cartilage (Figure 4B and D) and a large number of horizontal clefts in the junction between calcified and non-calcified cartilage (arrowheads in Figure 4), which is suggested to be closely related to the rapid degeneration and peeling of articular cartilage 23.

### Long term usage of Dex results in decrease subchondral bone mass and bone density

**Figure 5:**
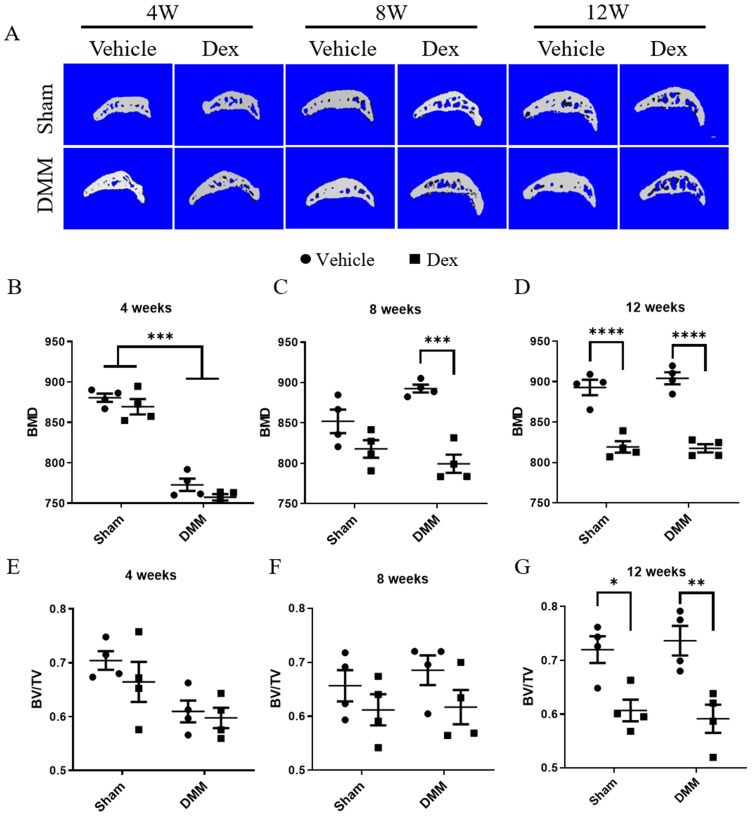
**Micro-CT analysis the effects of long-term usage of Dex on subchondral bone after DMM surgery. (A)** Micro-CT 3D images of subchondral bone in tibia at 4, 8 and 12 weeks after DMM surgery with or without Dex. **(B-D)** Results showing relative bone mass density (BMD), measurement in the total subchondral bone of tibia with vehicle and Dex treatment after DMM or sham operation (n = 4), showing that the BMD was decreased of tibia at 12 weeks with or without DMM surgery. **(E-G)** Results showing relative bone volume fraction (bone volume/total volume, BV/TV), measurement in the total subchondral bone of tibia with vehicle and Dex treatment after DMM or sham operation, showing that the BV/TV was decreased of tibia at 12 weeks with or without DMM surgery (n = 4). Comparisons of multiple groups were evaluated using analysis of variance (ANOVA) followed by Tukey's test. Data were expressed as the mean ± SEM. *, P < 0.05; **, P < 0.01;***, P < 0.001; ****, P < 0.0001.

OA is considered as a disease of the whole joint 24. In recent years, a large number of studies have shown that dysregulated subchondral bone remodeling is also involved in OA process 25. Therefore, we further observed the effect of Dex on subchondral bone in DMM surgery model. In Figure 5A-G, at 4 weeks after DMM operation, the bone mineral density (BMD) of subchondral bone was lower in Dex-treated group than those in Vehicle group although without statistical significance. Besides, Dex-treated resulted in the decreased BMD of subchondral bone with Dex treatment became more profound in mice at 8 weeks after DMM surgery. However, there were no significant differences in bone mass/volume ratio (BV/TV) of subchondral bone between sham and DMM group with/without Dex measured at 4 and 8 weeks. Consistently, repeated Dex injection resulted in decreased BMD and BV/TV of subchondral bone in both sham and DMM group at 12 weeks after DMM surgery. These results show that the systemic Dex application could lead to loss of subchondral bone mass and decreased bone density, which was consistent with previous study 23.

### Dex induces extracellular matrix calcification of chondrocytes through activation of AKT signaling

**Figure 6:**
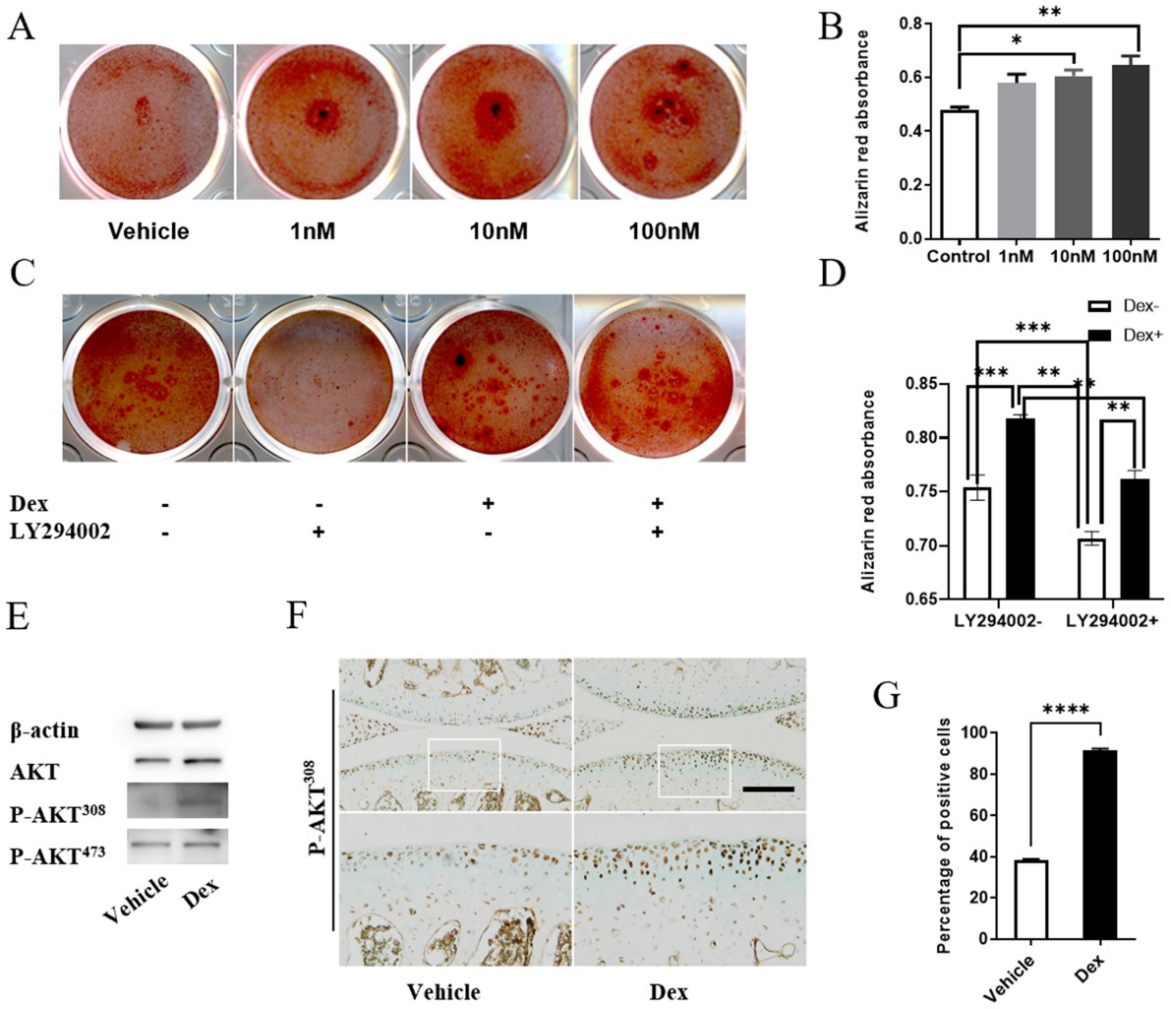
** Dex induces calcification of ECM partially by activating AKT signaling. (A)** Alizarin red staining to assess the extent of extracellular matrix calcification of Dex at different concentration in primary chondrocytes. **(B)** Alizarin red absorbance was detected by microplate reader at 562 nm (n = 3). **(C)** Alizarin red staining to assess the extent of extracellular matrix calcification of 10 nM Dex with or without AKT inhibitor LY294002 in primary chondrocytes. **(D)** Alizarin red absorbance was detected by microplate reader at 562 nm (n = 6). **(E)** Cell lysates of primary chondrocytes were analyzed by western blotting using antibodies of AKT, P-AKT^308^, P-AKT^473^ (n = 3). **(F)** IHC analysis of P-AKT^308^ protein expression in articular cartilage of mice at 4 weeks after DMM surgery with or without Dex, expansion of the region occupied by articular cartilage, Scale bar: 100 µm. **(G)** The percentage of cells that are positive for P-AKT^308^ in the articular cartilage were calculated (n = 4). Differences between two groups were evaluated using Student's unpaired *t*-test, and comparisons of multiple groups were evaluated using analysis of variance (ANOVA) followed by Tukey's test. Data were expressed as the mean ± SEM. *, P < 0.05; **, P < 0.01; ***, P < 0.001; ****, P < 0.0001.

Previous study showed that AKT could positively regulate the calcification of chondrocytes *in vitro*, and calcified osteophyte formation was prevented in the joints of Akt1 knockout mice with surgically induced OA 26. Thus, we examined whether modulation of AKT signaling affect the extracellular matrix calcification of cultured chondrocytes with or without Dex treatment. Firstly, we studied the effects of Dex at different concentrations (0, 1, 10, 100 nM) on the extracellular matrix calcification in primary chondrocytes. Alizarin red staining showed that Dex promoted the calcification of extracellular matrix of chondrocytes in a dose-dependent manner (Figure 6A), which was proved by the quantitative data present as OD562 (Figure 6B). While inhibition of AKT signaling by inhibitor LY294002 can reverse the Dex-induced increase of extracellular matrix calcification of chondrocytes (Figure 6C-D), suggesting that AKT signaling pathway plays an important role in Dex-mediated calcification of extracellular matrix of chondrocytes *in vitro*. Next, we examined whether Dex could affect the protein level of phosphorylated AKT. Western blotting result showed that Dex treatment increased the levels of P-AKT^308^ instead of that of P-AKT^473^ in cultured chondrocytes (Figure 6E), suggesting that Dex may partially lead to calcification of extracellular matrix of chondrocytes through activation of P-AKT^308^. Subsequently, we further examined the activation state of AKT in the joint sample from Dex-treated mice. IHC result showed that the number of P-AKT^308^ positive cells was significantly increased in articular cartilage (Figure 6F), and 90% of cells were positive for P-AKT^308^, which were mainly located in the deep layer of cartilage in Dex-treated group (Figure 6G). These results suggest that Dex may partially, at the early stage, activate the AKT signaling pathway to cause abnormal calcium deposition in calcified cartilage layer of OA cartilage.

### Dex promotes the apoptosis of articular chondrocytes *in vivo*

**Figure 7:**
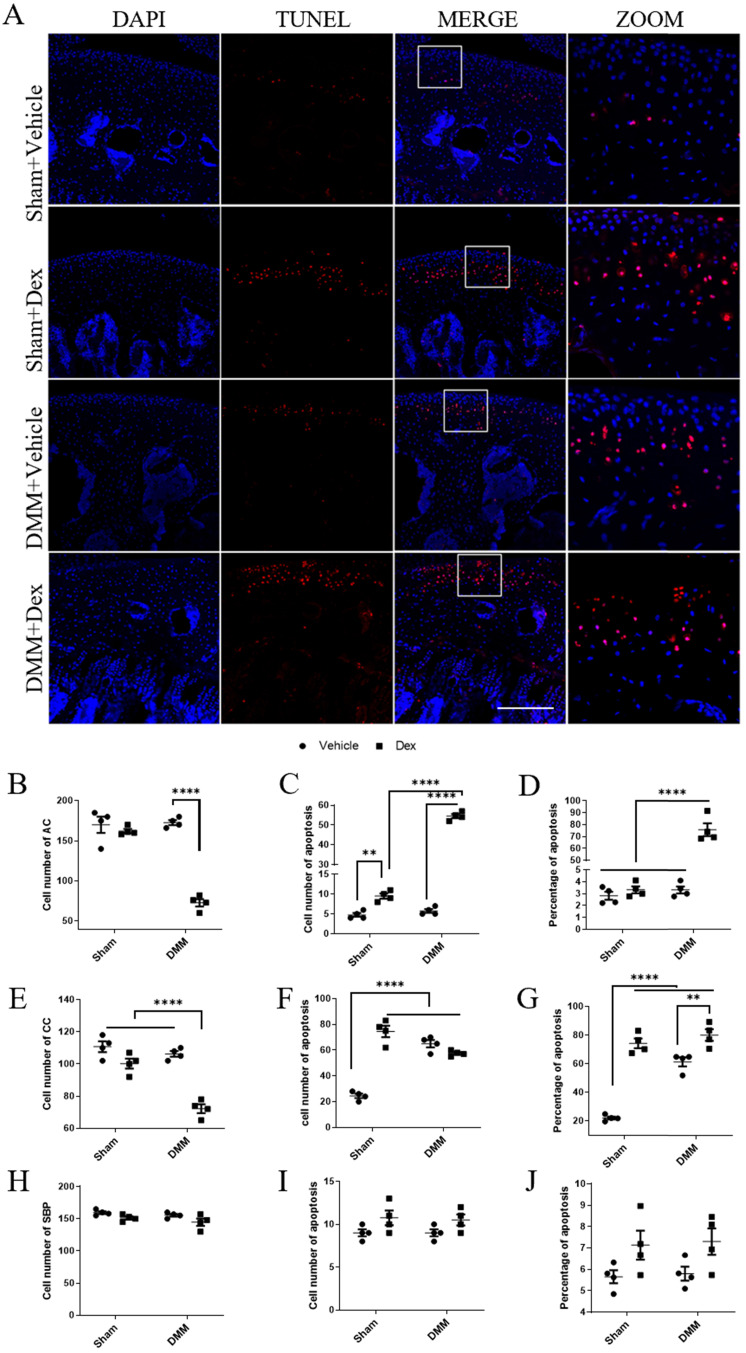
** Effects of long term usage of Dex on chondrocyte apoptosis in mice at 8 weeks after DMM. (A)** TUNEL assay was performed on knee joints to measure chondrocyte apoptosis at 8 weeks either sham operation or after DMM surgery with or without long term usage of Dex. **(B-J)** The total number of cells (B, E, H), the number of apoptotic cells (C, F, I) and the proportion of apoptotic cells (D, G, J) in non-calcified cartilage layer (B-D), calcified cartilage layer (E-G) and subchondral bone plate (H-J) were calculated (n = 4). Scale bar: 500 µm. Comparisons of multiple groups were evaluated using analysis of variance (ANOVA) followed by Tukey's test. Data were expressed as the mean ± SEM. *, P < 0.05; **, P < 0.01; ****, P < 0.0001.

Chondrocyte apoptosis is believed to play an important role in the pathogenesis of OA 27. Previous studies have revealed that Dex could enhance apoptosis in multiple types of cells 28, 29. Therefore, we further estimated the effect of Dex on the apoptosis of articular chondrocytes in sham and DMM mice. For the sham group, the number of cells were not changed significantly in the Vehicle group and the Dex group (Figure 7 B, E, H), while the number of apoptotic cells in the non-calcified layer increased significantly compared with the Vehicle group (Figure 7C), and the proportion of apoptotic cells did not change significantly (Figure 7D). But in the calcified layer, the number and proportion of apoptotic cells were significantly higher than those in Vehicle group (Figure 7F-G). Similarly, in the DMM + Vehicle group, we also found that apoptotic cells were relatively concentrated in the CCL (Figure 7F). But in the DMM + Dex group, the number of chondrocytes in both non-calcified layer and CCL were significantly decreased (Figure 7B, E). Both the number and proportion of apoptotic cells were increased in the non-CCL (Figure 7C-D). While the number of apoptotic cells in the CCL was not significantly changed, and the proportion of apoptotic cells was significantly increased due to the decreased total number of cells (Figure 7F-G). These results suggest that systemic use of Dex promotes apoptosis of articular chondrocytes, mainly in deep layers, and aggravates full-thickness chondrocyte apoptosis in experimental mouse OA model.

### Dex weakens the stress-response autophagy with the passage of time

**Figure 8:**
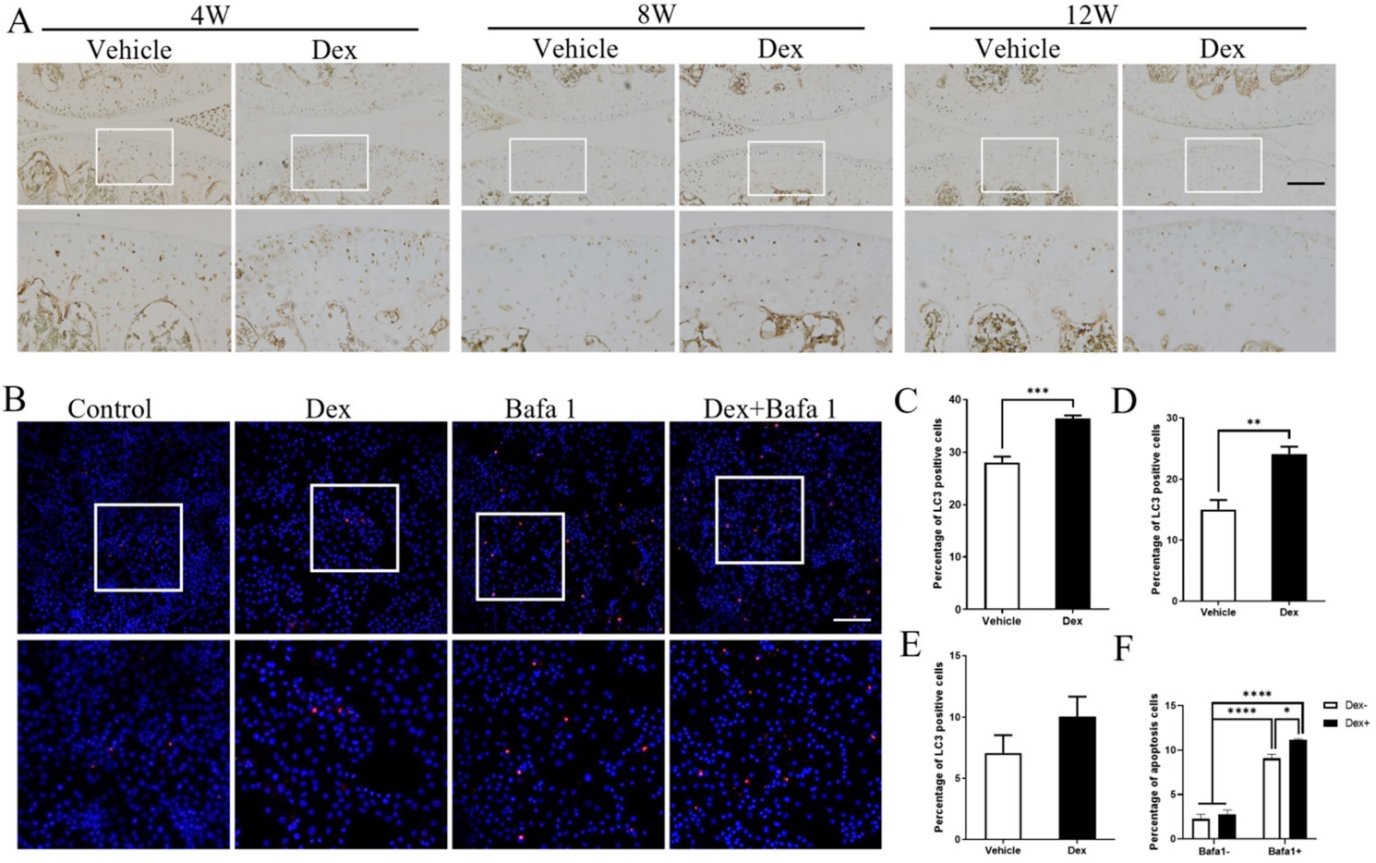
** Effects of repeated Dex on LC3 expression in articular cartilage in DMM mice model. (A)** The effect of Dex on LC3 expression in articular cartilage of DMM mice was analyzed by IHC. **(B)** TUNEL assay was used to analyze the effect of autophagy inhibitor Baf-A1 on the apoptosis of primary chondrocytes with or without Dex, the percentage of apoptosis cells in the articular cartilage were calculated (n = 3). Scale bar: 100 µm. **(C-E)** Quantification of cells positive for LC3 in articular cartilage of vehicle and Dex group at 4 weeks (B), 8 weeks (C) and 12 weeks (D) after DMM surgery, respectively (n = 4). **(F)** Quantification of apoptotic cells in cultured primary chondrocytes (n = 3). Differences between two groups were evaluated using Student's unpaired *t*-test, and comparisons of multiple groups were evaluated using analysis of variance (ANOVA) followed by Tukey's test. Data were expressed as the mean ± SEM. *, P < 0.05; **, P < 0.01; ***, P < 0.001; ****, P < 0.0001.

As autophagy could protect the cells from apoptosis in various conditions 30, and Dex was reported to induce autophagy in cultured chondrocytes 31, we deduced whether autophagy is involved in the Dex-induced chondrocyte apoptosis in DMM model. Our results showed that the numbers of LC3-positive cells in articular cartilage were significantly higher in Dex group than those in the Vehicle group at 4 weeks and 8 weeks following DMM surgery (Figure 8A, C and D). In addition, at 12 weeks after DMM operation, the number of LC3-positive cells were higher in Dex-treated group than those in Vehicle group although without statistical significance (Figure 8A and E). Furthermore, we performed TUNEL assay to analyze the effect of Dex-induced autophagy of chondrocytes with/without autophagy inhibitor Baf-A1. Our results showed that Baf-A1 could enhance the apoptosis induced by Dex alone in primary chondrocytes (Figure 8B and F). These results demonstrate that Dex could induce a protective autophagy, and the effect of stress-response autophagy was eliminated with the passage of time.

## Discussion

OA is the most common joint disease, affecting an estimated 10% of men and 18% of women over 60 years of age 8, which is currently accepted as a whole joint disease 25 and even whole-person disease 32 with irreversible process. The epidemiology 33 of this disorder is complex and multifactorial, with genetic, biological, and biomechanical components. However, there is still lack of disease-modifying treatment. An improved knowledge of the pathogenesis and further study of the risk factors of OA are helpful to deepen the understanding of OA pathogenesis and provide the basis for developing new prevention and treatment strategies. Dex is a widely used synthetic glucocorticoid 9. Due to the large number of patients with OA and long-term users of Dex, there are large number of OA patients using Dex. It's very necessary to study the effects of systemic application of glucocorticoids on articular cartilage homeostasis and OA pathogenesis.

In this study, we explored the effect of long-term administration of Dex on the homeostasis of articular cartilage and the progression of OA in mouse models. The effect of glucocorticoids has been previously studied mostly in cultured chondrocytes. In cultured rat articular chondrocytes, Dex has been shown to cause a decrease expression of type II collagen 34. After Dex treatment, the level of aggrecan expression was reduced in chondrocytes isolated from human OA cartilage samples 35. In current *in vivo* study, we demonstrated that Dex aggravates the loss of proteoglycan content of articular cartilage in adult mice. In addition, we found that Dex exacerbates the pathological severity in experimental OA model of mice. These data indicate that long-term administration of Dex may disturb the homeostasis of articular cartilage.

The articular cartilage can be divided into contain non-calcified layer (AC) and calcified cartilage layer (CCL), which is separated by the tidemark. The calcified cartilage zone borders is highly interdigitated with the subchondral bone 36. Among them, the CCL forms an important interface between compliant cartilage and stiff bone by transmitting force, attaching cartilage to bone, and limiting materials diffusion 37. Contributing to the stiffness gradient in the soft-hard tissue junction, the calcified cartilage zone is about 100 times stiffer than the overlying hyaline cartilage and 10 times less stiff than the underlying bone 38. However, this stiffness gradient is altered in association with degenerative change, the increased stiffness of calcified cartilage layer tends to subchondral bone in the OA condition 39. Our data clearly showed that the increased calcium content in the CCL from 4 weeks to 12 weeks after Dex treatment. Besides, Dex group showed a suddenly abnormal increase of calcium content in the CCL at the 4 weeks in experimental OA model of mice. Moreover, the samples from OA patients with a history of Dex treatment presented a high calcium content in calcified cartilage. These results were consistent with previous study 40, which have found that extremely enhanced hyper-mineralization was found in calcified cartilage zone in OA. For calcified cartilage, the nanoindentation modulus is positively related to the local mineral content 40, 41, which means the mechanical properties of cartilage may be affected by the extent of its mineralization. Furthermore, systemic application of Dex lead to loss of subchondral bone mass and decreased bone density. These data indicate that Dex may disturb the mechanical properties of cartilage via its modulation of the calcium content of CCL, as well as subchondral bone mass and bone density.

AKT plays an important role in the maintenance of cartilage homeostasis and the progression of OA. Calcified osteophyte formation was prevented in Akt1 knockout mice with surgically induced OA. Calcification was suppressed in cultured Akt1 deficiency chondrocytes or ATDC5 cells with Akt1 knockdown but enhanced in ATDC5 cells overexpressing constitutively active Akt1 26. In current study, we found that inhibition of AKT signaling by inhibitor LY294002 can ameliorate the Dex-induced increase of extracellular matrix calcification in cultured chondrocytes, suggesting that Dex lead to calcification of extracellular matrix of chondrocytes partially through activation of AKT. Akt signaling pathway plays an important role in cartilage degeneration through decrease the autophagy activity of chondrocytes 42. Intra-articular injection of Mg^2+^ alleviates extracellular matrix calcification and protects articular cartilage by inhibiting the autophagy formation 43. Whether Akt-mediated autophagy is involved in the Dex induced extracellular matrix mineralization of chondrocytes need to be further investigated. Furthermore, we showed that Dex promotes the apoptosis of articular chondrocytes *in vivo*. Chondrocyte apoptosis is generally observed prior to the transition of calcified cartilage to bone; however, chondrocyte apoptosis is not essential for cartilage calcification 44. Whether apoptosis is related to mineralization of chondrocytes ECM during OA development remains to be further studied. AKT signaling pathway is highly associated with chondrocyte apoptosis. Previous study has found that IL-1β-mediated activation of NF-κB and apoptosis in chondrocytes was inhibited by IGF-1 and PDGF-bb, which could be related to the suppression of Src/PI-3K/AKT pathway 45, which is consistent with our current conclusion. However, the constitutively active AKT rescued the expression of phenotypic markers and the apoptosis induced by CXCR2 blockade, indicating CXCR2-dependent chondrocyte homeostasis was mediated by AKT signaling 46. These data indicate that the AKT and its downstream signaling pathways may be involved in the maintenance and biological functions of chondrocytes after long term application of Dex, however, the detailed roles are needed to be studied.

Autophagy contributes to the maintenance of homeostasis of chondrocytes, whose impairment greatly aggravates OA 47. Cartilage-specific ablation of mTOR results in increased autophagy level and reduced articular cartilage degradation, apoptosis and synovial fibrosis in experimental OA model 48. In this study, Dex induced a protective autophagy of chondrocytes at the early stage, which was gradually weakened with the extension of processing time. Besides, we found that inhibition of autophagy by inhibitor Bafa1 can enhance the apoptosis induced by Dex in primary chondrocytes. These data indicate that autophagy may be one of the adaptive protective responses for chondrocytes under the stimuli of Dex. Long-time application of Dex, however, weakened the autophagy-mediated protective effect and promoted chondrocyte apoptosis, ultimately aggravated the damage of cartilage. Thus, future studies on the role and underlying mechanisms of activation or inhibition of autophagy of chondrocytes using autophagy-related mouse model will provide more insights.

Taken together, our findings suggest that long term usage of Dex accelerating the initiation of OA via enhancing the extracellular matrix calcification and apoptosis of chondrocytes, which will help to optimize the safe use of Dex in the clinic as a disease-modifying drug.
